# The efficient synthesis of dibenzo[*d*,*d*′]benzo[1,2-*b*:4,3-*b*′]dithiophene and cyclopenta[1,2-*b*:4,3-*b*′]bis(benzo[*d*]thiophen)-6-one

**DOI:** 10.3762/bjoc.5.55

**Published:** 2009-10-13

**Authors:** Zhihua Wang, Sheng Zhu, Jianwu Shi, Hua Wang

**Affiliations:** 1 Key Lab for Special Functional Materials of Ministry of Education, Henan University, Kaifeng 475004, China; 2 College of Chemistry and Chemical Engineering, Henan University, Kaifeng 475004, China

**Keywords:** crystal structure, cyclopenta[1,2-*b*:4,3-*b*′]bis(benzo[*d*]thiophen)-6-one, dibenzo[*d*,*d*′]benzo[1,2-*b*:4,3-*b*′]dithiophene, McMurry reaction, preparation

## Abstract

With 3,3′-bi[benzo[*b*]thiophenyl] as starting material, dibenzo[*d*,*d*′]benzo[1,2-*b*:4,3-*b*′]dithiophene, a [5]heterohelicene, was synthesized efficiently in 60% yield via formylation and McMurry reaction. Cyclopenta[1,2-*b*:4,3-*b*′]bis(benzo[*d*]thiophen)-6-one, another interesting helical ketone, was also prepared in 79% yield via deprotonation and ketonization of 3,3′-bi[benzo[*b*]thiophenyl]. In addition, the single-crystal structure of dibenzo[*d*,*d*′]benzo[1,2-*b*:4,3-*b*′]dithiophene and UV–vis spectra of both title compounds are described.

## Introduction

With considerable environmental stability and flexibility in synthesis, π-extended heteroarenes containing thiophene rings within an polyaromatic ring system are currently of great interest because they can potentially be used to fabricate organic field-effect transistors (OFETs) [1–7], light emitting diodes (LEDs) [8–9], and photovoltaic cells [10]. Various novel π-extended heteroarenes, e.g. dibenzo[*d*,*d*′]thieno[3,2-*b*:4,5-*b*′]dithiophene [11], pentathienoacene [12], and benzo[1,2-*b*:4,5-*b*′]bis[*b*]benzothiophene [13], have been developed and tested as active semiconducting channels in OFET devices due to their structural resemblance to pentacene [14] which possesses very high field-effect mobility (~3.0 cm^2^ V^−1^ s^−1^) for OFET devices. However, one of the possible analogs, dibenzo[*d*,*d*′]benzo[1,2-*b*:4,3-*b*′]dithiophene (**1**), an interesting [5]heterohelicene, has not received much attention during the last decade because of a lack of efficient synthesis, which limits its application in material science.

**Figure 1:**
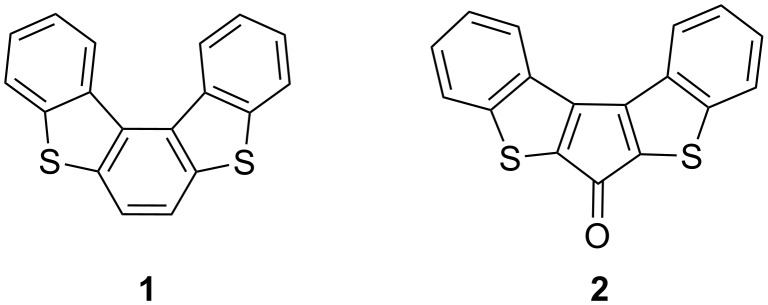
Chemical structures of **1** and **2**.

Photocyclization is normally employed for preparing heterohelicenes with 1,2-diaryl ethenes as precursors. Wynberg et al. [15] reported the synthesis of **1** in 57% yield by the photocyclization of 1,2-di(2-benzo[*b*]thienyl)ethene in benzene. Kudo et al. [16] improved the preparative yield of **1** up to 85% with the same precursor in the same way. Sugimoto et al. [17] reported the preparation of **1** in 19% yield via the photolysis of RCH(SPh)_2_ (R = benzo[*b*]thiophene). However, a non-photochemical method for preparing **1** has, to the best of our knowledge, not been reported. In our work, with 3,3′-bis[benzo[*b*]thiophenyl] (**3**) [18] as starting material, **1** (see Figure 1) was synthesized efficiently via formylation and McMurry reaction and its crystal structure was determined. In addition, cyclopenta[1,2-*b*:4,3-*b*′]bis(benzo[*d*]thiophen)-6-one (**2**), an interesting helical ketone, was also prepared via deprotonation and ketonization of **3**.

## Results and Discussion

### Synthesis

**Scheme 1:**
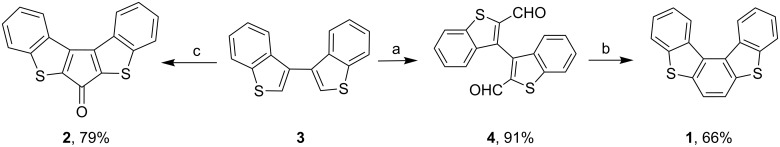
Synthetic route to **1** and **2**. *Reagents and conditions*: (a) (i) *n*-BuLi (2.3 equiv), Et_2_O, reflux; (ii) −78 °C, DMF (4.0 equiv); (b) TiCl_4_ (3.0 equiv), Zn (6.0 equiv), pyridine (3.0 equiv), THF, reflux; (c) (i) *n*-BuLi (2.3 equiv), Et_2_O, reflux; (ii) Me_2_NCOCl (1.0 equiv), −50 °C to RT.

Scheme 1 shows the synthetic route to **1** and **2**. With **3** as starting material, formylation and McMurry reaction were employed for preparing **1**. The formylation step involves sequential deprotonation of **3** and treatment of the intermediate dilithiated species with *N*,*N*-dimethylformamide (DMF). Attempted generation of dilithiated **3** with either lithium diisopropylamide (LDA) or *n*-BuLi at −78 °C failed. However, under refluxing conditions, direct deprotonation of **3** with 2.3 equiv *n*-BuLi in anhydrous Et_2_O was successful. After quenching with excess DMF, [3,3′]bi[benzo[*b*]thiophenyl]-2,2′-dicarbaldehyde (**4**) was obtained in high yield (91%). The next step is the intramolecular McMurry reaction of **4**. We found that the reactivity of elemental titanium is crucial in this step and that the Ti(0) must be freshly prepared from zinc dust, pyridine and TiCl_4_ in THF. By following standard procedures [19–21], the title compound **1** was obtained in 66% yield. The overall yield of **1** from **3** is about 60%. When dilithiated **3** was treated with *N*,*N*-dimethylcarbamoyl chloride (DMC) at low temperature (−50 °C), a new helical ketone, cyclopenta[1,2-*b*:4,3-*b*′]bis(benzo[*d*]thiophen)-6-one (**2**), was obtained in 79% yield.

### X-ray structural analysis

**Figure 2:**
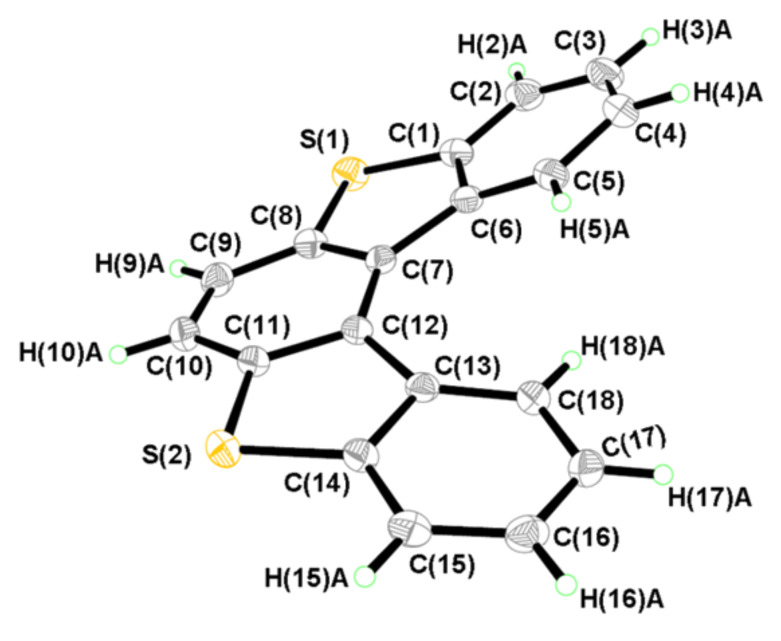
Molecular structure and conformation of **1**.

**Figure 3:**
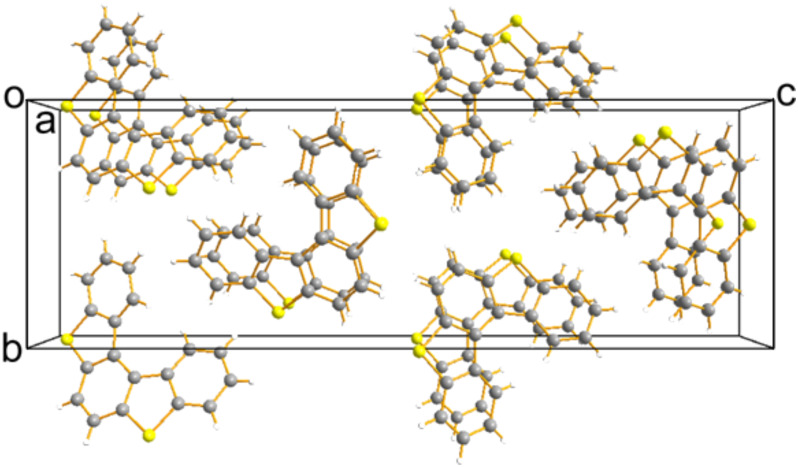
Crystal packing structure of **1**.

A single crystal of **1** was obtained by the slow evaporation of a solution of **1** in CHCl_3_/CH_3_OH (5/1, v/v). The crystal structure of **1** was confirmed by single-crystal X-ray analysis (Figure 2 and Figure 3). **1** has non-planar π-extended frameworks, and its molecule is compressed and dominated by a helical structure (Figure 2). The distance between the two H atoms H(5)A…H(18)A is 2.062 Å and these H atoms point away from each other. The repulsion of the facing terminal benzene rings causes an interplanar angle of 31.7° between the terminal benzene rings. The angles between the least-squares planes of neighboring rings are between 7.5° and 9.5°. Using the middle benzene ring as a reference, the inner (C(5), C(6), C(7), C(12), C(13), and C(18)) helix increases by 1.46 Å and turns in-plane by 237.0° [22–23].

The crystal packing structure of **1** (Figure 3) is based on π-stacking along the *a*-axis in which the plane-to-plane distance is ca. 3.857 Å. The lack of contact between the π-stacks indicates that the crystal has one-dimensional (1D) electronic structure [13].

### UV–vis spectra of **1** and **2**

**Figure 4:**
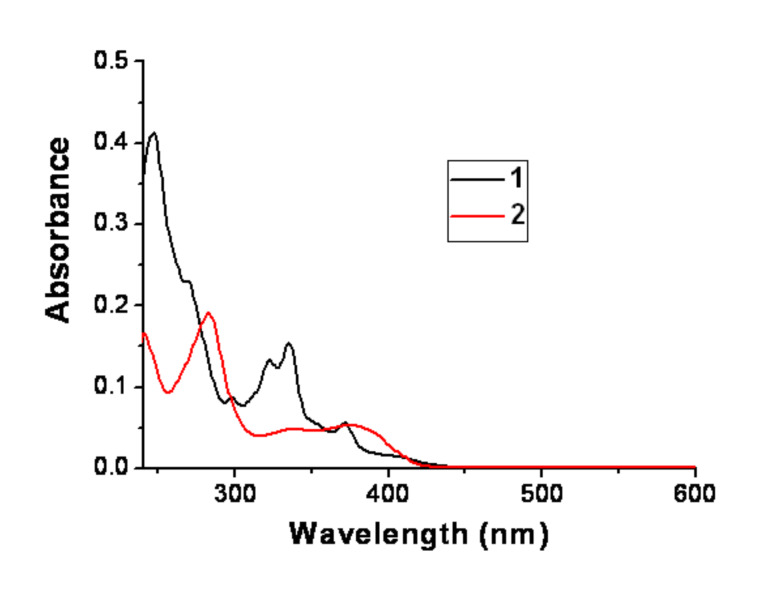
UV–vis spectra of **1** and **2** in chloroform ([*C*] = 1 × 10^−5^ M).

The UV–vis spectra of **1** and **2** in chloroform are shown in Figure 4. **1** shows the main peaks at 323 nm, 335 nm, and 372 nm, and **2** gives two broad peaks at 339 nm and 380 nm. Both **1** and **2** possess molecular connectivities of cross-conjugated π-system that shows π-electron delocalization with helical distortion.

## Conclusion

Derivatives of benzothiophene, thienothiophene, and thienodithiophene with good characteristics for OFETs were recently reported [5–7,11,24–25]. Because of higher π-electron delocalization, compounds **1** and **2** could be used in OFET and/or conducting polymers. From **3** as starting material, **1** and **2** have been efficiently obtained in a total yield of 60% and 79%, respectively, in our work. The efficient synthesis of **1** and **2** will facilitate the synthetic approaches to various organic functional materials, by using **1** and **2** as building blocks, and/or versatile intermediates. The measurement of the hole/electron mobilities of compounds **1** and **2** is in progress.

## Experimental

### Synthesis of [3,3′]bi[benzo[*b*]thiophenyl]-2,2′-dicarbaldehyde (**4**)

To a solution of **3** (0.2986 g, 1.12 mmol) in anhydrous Et_2_O (30 mL), *n*-BuLi (1.0 mL, 2.58 mmol, 2.3 equiv) was added dropwise at −78 °C. The resulting reaction mixture was slowly warmed to ambient temperature, then heated to 50 °C, refluxed for 2 h, and quenched with dry DMF (0.35 mL, 4.48 mmol, 4.0 equiv) at −78 °C. The reaction mixture was warmed slowly to ambient temperature overnight. The reaction mixture was quenched with H_2_O, extracted with Et_2_O (3 × 15 mL), washed with H_2_O (2 × 30 mL), and dried over MgSO_4_. The residue was purified by column chromatography on silica gel with PE (60–90 °C)/ethyl acetate (8/1, v/v) as eluent to yield **4** (0.3284 g, 90.8%) as light yellow crystals. mp 143–144 °C; ^1^H NMR (400 MHz, CDCl_3_): δ 9.82 (s, 2H), 8.03 (d, *J* = 8.4 Hz, 2H), 7.64–7.54 (m, 4H), 7.42 (t, *J* = 7.6 Hz, 2H); ^13^C NMR (100 MHz, CDCl_3_): δ 184.2, 141.9, 141.7, 139.6, 136.6, 129.1, 126.0, 125.0, 123.5. HRMS (TOF MS EI^+^) *m*/*z*: calcd for [C_18_H_10_O_2_S_2_] 322.0122, found 322.0126. IR (KBr): 2928, 2823 (C–H), 1668 (C=O) cm^−1^.

### Synthesis of dibenzo[*d*,*d*′]benzo[1,2-*b*:4,3-*b*′]dithiophene (**1**)

TiCl_4_ (0.16 mL, 1.5 mmol, 3.0 equiv) was carefully added to dry THF (20 mL) at 0 °C, after keeping at 0 °C for 10 min, zinc dust (0.20 g, 3.0 mmol, 6.0 equiv) was added, and the mixture refluxed for 2 h. Pyridine (0.12 mL, 1.5 mmol, 3.0 equiv) was added and the mixture was heated under reflux for further 1 h. After cooling to ambient temperature, a solution of **4** (0.1607 g, 0.5 mmol) in dry THF (10 mL) was added and the reaction mixture was heated under reflux overnight. The reaction mixture was quenched with 18% HCl (20 mL) at 0 °C, extracted with CHCl_3_ (4 × 30 mL), washed with H_2_O (3 × 30 mL), and dried over MgSO_4_. After the removal of the solvent in vacuo, the crude product was purified by column chromatography on silica gel with PE (60–90 °C)/ethyl acetate (8/1, v/v) as eluent to yield **1** (0.0958 g, 66.2%) as a white solid. mp 182–183 °C (lit. mp 183–185 °C [15], 184–185 °C [16]); ^1^H NMR (400 MHz, CDCl_3_): δ 9.05–8.99 (m, 2H), 8.02–7.95 (m, 2H), 7.91 (s, 2H), 7.56–7.50 (m, 4H); ^13^C NMR (100 MHz, CDCl_3_): δ 139.9, 137.3, 135.2, 131.2, 126.3, 125.0, 123.5, 123.1, 121.1. HRMS (TOF MS EI^+^) *m*/*z*: calcd for [C_18_H_10_S_2_] 290.0224, found 290.0226. IR: 3054, 2922, 2852 (C–H) cm^−1^.

### Synthesis of cyclopenta[1,2-*b*:4,3-*b*′]bis(benzo[*d*]thiophen)-6-one (**2**)

To a solution of **3** (0.1556 g, 0.58 mmol) in anhydrous Et_2_O (40 mL), *n*-BuLi (0.4 mL, 1.23 mmol, 2.1 equiv) was added dropwise at −78 °C. The resulting reaction mixture was slowly heated to 50 °C and refluxed for 2 h. After being cooled to −50 °C, DMC (0.05 mL, 0.58 mmol, 1.0 equiv) was added dropwise. The reaction mixture was warmed slowly to ambient temperature overnight. The reaction mixture was quenched with saturated NH_4_Cl (20 mL) at 0 °C, extracted with Et_2_O (3 × 20 mL), washed with H_2_O (3 × 30 mL), and dried over MgSO_4_. The residue was purified by column chromatography on silica gel with CHCl_3_ as eluent to yield **2** (0.1356 g, 79.4%) as orange crystals. mp 180 °C (decomposed); ^1^H NMR (400 MHz, CDCl_3_): δ 8.23 (d, *J* = 8.0 Hz, 2H), 7.84 (d, *J* = 8.0 Hz, 2H), 7.51 (td, *J* = 7.6, 1.2 Hz, 2H), 7.40 (td, *J* = 7.6, 1.2 Hz, 2H). HRMS (TOF MS EI^+^) *m*/*z*: calcd for [C_17_H_8_OS_2_] 290.0017, found 290.0020. IR (KBr): 3053, 2927 (C–H), 1709 (C=O) cm^−1^.

### Crystal data for **1**

*M* = 290.38, C_18_H_10_S_2_, orthorhombic, space group *P*2(1)2(1)2(1), *a* = 3.9971(16) Å, *b* = 10.334(4) Å, *c* = 31.473(13) Å, α = 90°, β = 90°, γ = 90°, *V* = 1300.1(9) Å^3^, *Z* = 4, *d*_calcd_ = 1.484 g/cm^3^. A colorless crystal with a size of 0.29 mm × 0.15 mm × 0.10 mm was used for measurement at 296(2) K in ω scan mode (Bruker Smart APEX X-ray diffractometer, CCD detector, Mo K_α_ radiation (λ = 0.71073 Å)). The data were corrected for Lorentz and polarization effects and absorption corrections were performed using the SADABS [26] program. The crystal structures were solved using the SHELXTL [27] program and refined using full matrix least-squares. The positions of hydrogen atoms were calculated theoretically and included in the final cycles of refinement in a riding model along with attached carbons. The final cycle of full matrix least-squares refinement was based on 7988 independent reflections [*I* > 2σ(*I*)] and 181 variable parameters with *R*1 = 0.0335, *wR*2 = 0.0846.

## Supporting Information

Supporting information features experimental procedures and spectroscopic analysis for compounds **1**, **2**, and **4**.

File 1 The efficient synthesis of dibenzo[*d*,*d*′]benzo[1,2-*b*:4,3-*b*′]dithiophene and cyclopenta[1,2-*b*:4,3-*b*′]bis(benzo[*d*]thiophen)-6-one.

File 2 Crystal structure of compound **1** in cif format.
