# Supplementary material for: The efficient synthesis of dibenzo[d,d′]benzo[1,2-b:4,3-b′]dithiophene and cyclopenta[1,2-b:4,3-b′]bis(benzo[d]thiophen)-6-one
Source: Beilstein J Org Chem. 2009 Oct 13;5:55. doi: 10.3762/bjoc.5.55 (PMC2814327; doi:10.3762/bjoc.5.55)
Supplement: File 1 — The efficient synthesis of dibenzo[d,d′]benzo[1,2-b:4,3-b′]dithiophene and cyclopenta[1,2-b:4,3-b′]bis(benzo[d]thiophen)-6-one. [file Beilstein_J_Org_Chem-05-55-s001.doc]

# Supporting Information for

**The efficient synthesis of dibenzo[d,d′]benzo[1,2-b:4,3-b′]dithiophene and cyclopenta[1,2-b:4,3-b′]bis(benzo[d]thiophen)-6-one**

Zhihua Wang^1,2^, Sheng Zhu^1^, Jianwu Shi^1^, and Hua Wang^*1,2^

Address: ^1^Key Lab for Special Functional Materials of Ministry of Education, Henan University, Kaifeng 475004, China and ^2^College of Chemistry and Chemical Engineering, Henan University, Kaifeng 475004, China

Email: Hua Wang - [hwang@henu.edu.cn](mailto:hwang@henu.edu.cn)

* Corresponding author

**EXPERIMENTAL**

**General procedures and materials.** Ether and tetrahydrofuran (THF) for use were freshly distilled from sodium/benzophenone. n-BuLi (hexane) were from Acros or Alfa Aesar, its concentration was determined by titration with N-oivaloyl-o-toluidine. Column chromatography was carried out on silica gel (300–400 mesh). Standard techniques for synthesis under inert atmosphere, using gasbag and Schlenk glassware equipped with an 8-mm PTFE vacuum stop-cock (Synthware) were employed. All starting materials and reagents are commercially available.

^1^H and ^13^C NMR spectra were recorded on a Bruker AVANCE400M spectrometer. The chemical shift references were as follows: (^1^H ) CDCl_3_, 7.26 ppm (CHCl_3_); (^13^C) CDCl_3_, 77.0 ppm (CDCl_3_). IR spectra were obtained using an FT-IR instrument (Nicolet, AVATAR360), equipped with an ATR sampling accessory. UV–vis spectra were taken on a Lambda 35 (Perkin Elmer) spectrophotometer at room temperature. HRMS spectra were recorded on a mass spectrometer equipped with TOF (EI^+^) or TOF (CI^+^). Melting point determination was taken on a Melt-Temp apparatus (XT4-100) from *Beijing Keyi Electro-optic Instrument Plant* and were uncorrected.

**NMR spectra**

^1^H NMR (400 MHz, CDCl_3_) spectrum of **4**

^13^C NMR (100 MHz, CDCl_3_) spectrum of **4**

**
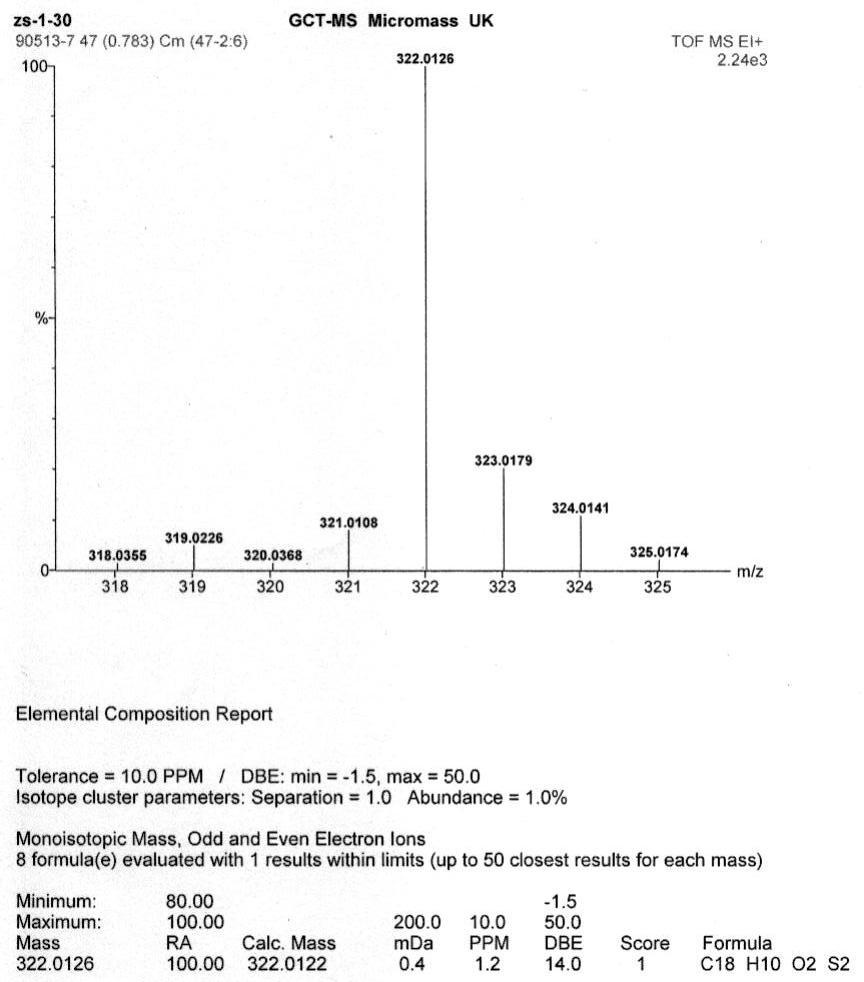
**

HRMS spectrum of **4**

^1^H NMR (400 MHz, CDCl_3_) spectrum of **1** (*****from petrol ether)

^13^C NMR (100 MHz, CDCl_3_) spectrum of **1**


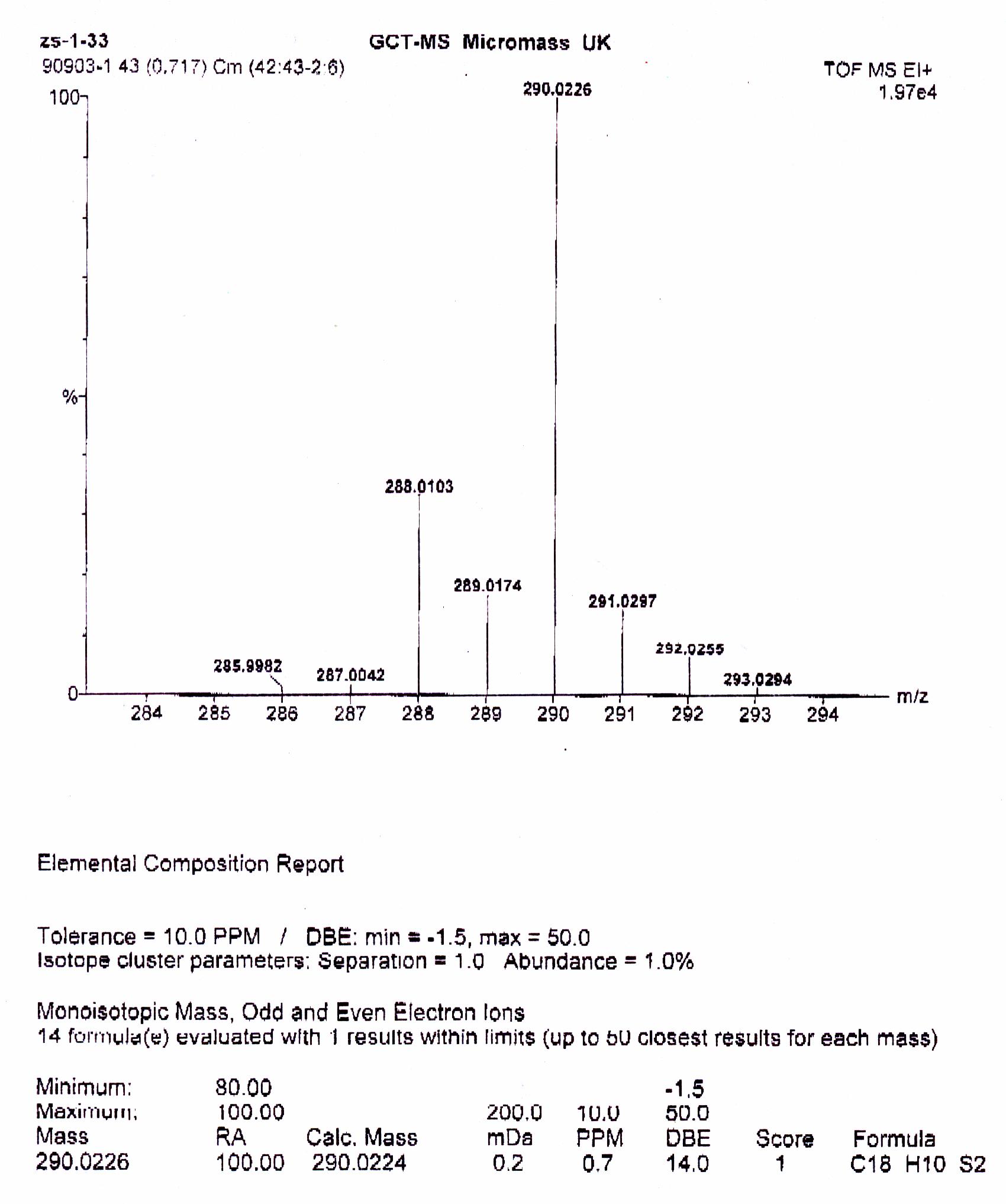


HRMS spectrum of **1**

^1^H NMR (400 MHz, CDCl_3_) spectrum of **2**


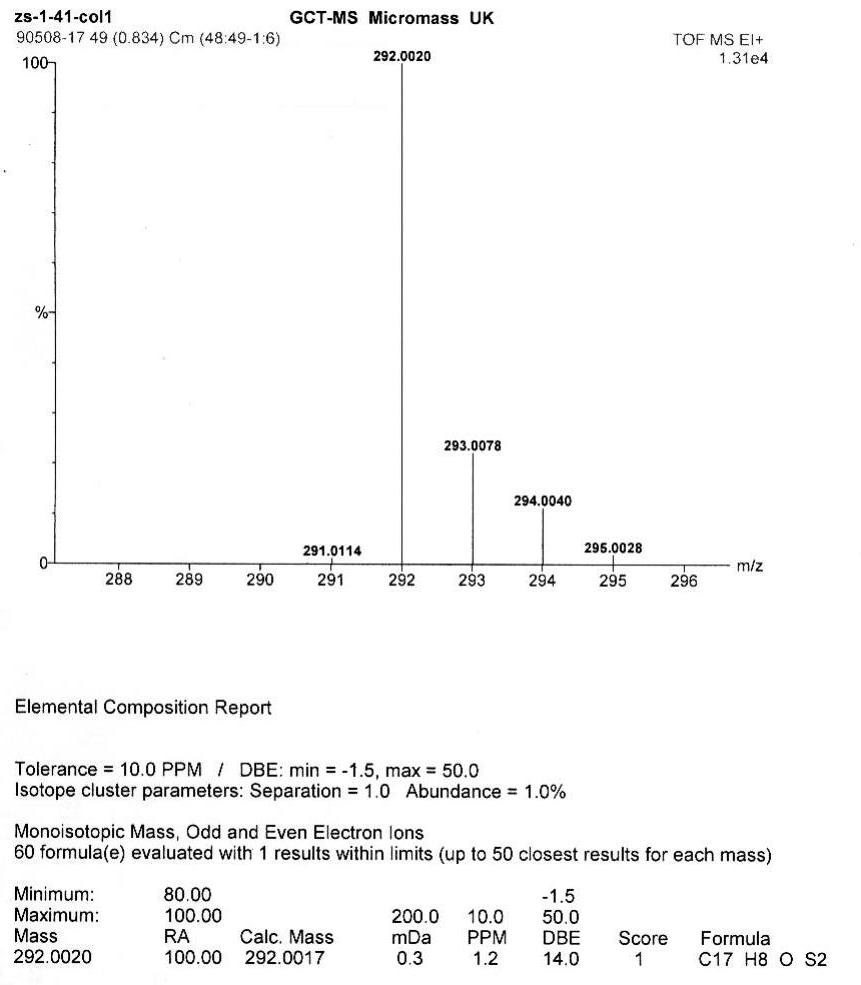


HRMS spectrum of **2**

**Crystal structure data for 1**

Table 1: Crystal data and structure refinement for **1**.

| Identification code | **1** |
| --- | --- |
| Empirical formula | C_18_H_10_S_2_ |
| Formula weight | 290.38 |
| Temperature | 296(2) K |
| Wavelength | 0.71073 Å |
| Crystal system, space group | Orthorhombic, P2(1)2(1)2(1) |
| Unit cell dimensions | a = 3.9971(16) Å α = 90°  b = 10.334(4) Å β = 90°  c = 31.473(13) Å γ = 90° |
| Volume | 1300.1(9) Å^3^ |
| Z, Calculated density | 4, 1.484 Mg/m^3^ |
| Absorption coefficient | 0.393 mm^−1^ |
| F(000) | 600 |
| Crystal size | 0.29 mm × 0.15 mm × 0.10 mm |
| Theta range for data collection | 1.29 to 28.20° |
| Limiting indices | −5<=h<=5, −13<=k<=10, −37<=l<=39 |
| Reflections collected/unique | 7988/3070 [R(int) = 0.0242] |
| Completeness to theta = 28.20 | 96.3% |
| Absorption correction | None |
| Max. and min. transmission | 0.9618 and 0.8946 |
| Refinement method | Full matrix least-squares on F^2^ |
| Data/restraints/parameters | 3070/0/181 |
| Goodness-of-fit on F^2^ | 1.042 |
| Final R indices [I > 2σ(I)] | R1 = 0.0335, wR2 = 0.0760 |
| R indices (all data) | R1 = 0.0395, wR2 = 0.0846 |
| Absolute structure parameter | 0.03(8) |
| Largest diff. peak and hole | 0.212 and −0.208 e. Å^−3^ |

Table 2: Atomic coordinates (×10^4^) and equivalent isotropic displacement parameters (A^2^ × 10^3^) for **1**. U(eq) is defined as one third of the trace of the orthogonalized U^ij^ tensor.

________________________________________________________________

x y z U(eq)

________________________________________________________________

S(1) 3338(2) 10025(1) 9752(1) 43(1)

S(2) 9646(2) 6468(1) 8373(1) 38(1)

C(12) 6929(5) 8631(2) 8664(1) 29(1)

C(13) 7140(5) 8777(2) 8203(1) 30(1)

C(11) 7962(6) 7375(2) 8788(1) 33(1)

C(7) 5762(5) 9477(2) 8990(1) 30(1)

C(6) 5185(6) 10881(2) 8997(1) 32(1)

C(15) 9058(6) 7597(2) 7569(1) 39(1)

C(18) 5767(6) 9725(2) 7934(1) 34(1)

C(14) 8646(6) 7685(2) 8009(1) 33(1)

C(16) 7826(6) 8574(2) 7317(1) 41(1)

C(1) 3778(6) 11288(2) 9387(1) 36(1)

C(8) 5090(6) 8930(2) 9391(1) 35(1)

C(5) 6142(6) 11843(2) 8707(1) 38(1)

C(3) 3874(7) 13497(2) 9168(1) 50(1)

C(9) 5895(6) 7643(2) 9495(1) 39(1)

C(17) 6132(6) 9617(2) 7496(1) 38(1)

C(2) 3041(7) 12592(2) 9468(1) 46(1)

C(10) 7471(6) 6886(2) 9201(1) 39(1)

C(4) 5469(7) 13137(2) 8794(1) 47(1)

________________________________________________________________

Table 3: Bond lengths [Å] and angles [°] for **1**.

| S(1)-C(1)  S(1)-C(8)  S(2)-C(11)  S(2)-C(14)  C(12)-C(11)  C(12)-C(7)  C(12)-C(13)  C(13)-C(18)  C(13)-C(14)  C(11)-C(10)  C(7)-C(8)  C(7)-C(6)  C(6)-C(5)  C(6)-C(1)  C(15)-C(16)  C(15)-C(14)  C(15)-H(15A)  C(1)-S(1)-C(8)  C(11)-S(2)-C(14)  C(11)-C(12)-C(7)  C(11)-C(12)-C(13)  C(7)-C(12)-C(13)  C(18)-C(13)-C(14)  C(18)-C(13)-C(12)  C(14)-C(13)-C(12)  C(10)-C(11)-C(12)  C(10)-C(11)-S(2)  C(12)-C(11)-S(2)  C(8)-C(7)-C(12)  C(8)-C(7)-C(6)  C(12)-C(7)-C(6)  C(5)-C(6)-C(1)  C(5)-C(6)-C(7)  C(1)-C(6)-C(7)  C(16)-C(15)-C(14)  C(16)-C(15)-H(15A)  C(14)-C(15)-H(15A)  C(17)-C(18)-C(13)  C(17)-C(18)-H(18A)  C(13)-C(18)-H(18A)  C(15)-C(14)-C(13)  C(15)-C(14)-S(2)  C(13)-C(14)-S(2)  C(15)-C(16)-C(17)  C(15)-C(16)-H(16A) | 1.748(2)  1.749(2)  1.744(2)  1.746(2)  1.417(3)  1.428(3)  1.461(3)  1.408(3)  1.417(3)  1.407(3)  1.407(3)  1.469(3)  1.404(3)  1.412(3)  1.375(3)  1.398(3)  0.9300  90.91(10)  90.93(10)  117.20(18)  110.62(18)  132.10(19)  117.44(18)  130.37(19)  111.70(18)  122.9(2)  123.54(16)  113.42(16)  117.47(18)  110.66(18)  131.84(19)  117.58(18)  130.3(2)  111.71(18)  118.8(2)  120.6  120.6  120.1(2)  120.0  120.0  121.9(2)  125.18(17)  112.85(15)  120.6(2)  119.7 | C(18)-C(17)  C(18)-H(18A)  C(16)-C(17)  C(16)-H(16A)  C(1)-C(2)  C(8)-C(9)  C(5)-C(4)  C(5)-H(5A)  C(3)-C(2)  C(3)-C(4)  C(3)-H(3A)  C(9)-C(10)  C(9)-H(9A)  C(17)-H(17A)  C(2)-H(2A)  C(10)-H(10A)  C(4)-H(4A)  C(17)-C(16)-H(16A)  C(2)-C(1)-C(6)  C(2)-C(1)-S(1)  C(6)-C(1)-S(1)  C(7)-C(8)-C(9)  C(7)-C(8)-S(1)  C(9)-C(8)-S(1)  C(4)-C(5)-C(6)  C(4)-C(5)-H(5A)  C(6)-C(5)-H(5A)  C(2)-C(3)-C(4)  C(2)-C(3)-H(3A)  C(4)-C(3)-H(3A)  C(10)-C(9)-C(8)  C(10)-C(9)-H(9A)  C(8)-C(9)-H(9A)  C(18)-C(17)-C(16)  C(18)-C(17)-H(17A)  C(16)-C(17)-H(17A)  C(3)-C(2)-C(1)  C(3)-C(2)-H(2A)  C(1)-C(2)-H(2A)  C(9)-C(10)-C(11)  C(9)-C(10)-H(10A)  C(11)-C(10)-H(10A)  C(3)-C(4)-C(5)  C(3)-C(4)-H(4A)  C(5)-C(4)-H(4A) | 1.390(3)  0.9300  1.392(3)  0.9300  1.404(3)  1.407(3)  1.391(3)  0.9300  1.370(4)  1.390(4)  0.9300  1.365(3)  0.9300  0.9300  0.9300  0.9300  0.9300  119.7  121.9(2)  125.15(18)  112.83(16)  122.9(2)  113.47(15)  123.53(17)  120.0(2)  120.0  120.0  120.9(2)  119.5  119.5  119.3(2)  120.3  120.3  120.9(2)  119.5  119.5  118.5(2)  120.7  120.7  118.9(2)  120.5  120.5  120.8(2)  119.6  119.6 |
| --- | --- | --- | --- |

Table 4: Anisotropic displacement parameters (Å^2^ × 10^3^) for **1**. The anisotropic displacement factor exponent takes the form: −2 π^2^ [ h^2^ a*^2^ U^11^ + ... + 2 h k a* b* U^12^].

____________________________________________________________

U^11^ U^22^ U^33^ U^23^ U^13^ U^12^

____________________________________________________________________

S(1) 48(1) 46(1) 34(1) −4(1) 6(1) −2(1)

S(2) 43(1) 29(1) 42(1) −1(1) −1(1) 5(1)

C(12) 27(1) 27(1) 33(1) 1(1) −3(1) −4(1)

C(13) 30(1) 28(1) 31(1) −2(1) −2(1) −5(1)

C(11) 33(1) 29(1) 38(1) −1(1) −5(1) −2(1)

C(7) 28(1) 31(1) 31(1) 0(1) −3(1) −3(1)

C(6) 32(1) 32(1) 33(1) −5(1) −5(1) −2(1)

C(15) 43(1) 36(1) 39(1) −8(1) 3(1) −2(1)

C(18) 36(1) 31(1) 36(1) 0(1) −2(1) −1(1)

C(14) 34(1) 30(1) 35(1) −1(1) 0(1) −3(1)

C(16) 48(1) 46(1) 30(1) −2(1) 1(1) −9(1)

C(1) 34(1) 36(1) 38(1) −6(1) −2(1) −2(1)

C(8) 36(1) 37(1) 33(1) −3(1) 0(1) −4(1)

C(5) 42(1) 33(1) 38(1) −2(1) −2(1) −5(1)

C(3) 51(2) 33(1) 67(2) −10(1) −11(1) 5(1)

C(9) 43(1) 41(1) 33(1) 7(1) −2(1) −4(1)

C(17) 43(1) 36(1) 36(1) 5(1) −4(1) −4(1)

C(2) 44(1) 45(1) 49(1) −14(1) −3(1) 4(1)

C(10) 42(1) 31(1) 43(1) 6(1) −7(1) −2(1)

C(4) 55(2) 34(1) 51(1) 1(1) −9(1) −6(1)

__________________________________________________________________

Table 5: Hydrogen coordinates (×10^4^) and isotropic displacement parameters (Å^2^ × 10^3^) for **1**.

________________________________________________________

x y z U(eq)

________________________________________________________________

H(15A) 10145 6891 7449 47

H(18A) 4614 10424 8048 41

H(16A) 8127 8537 7024 50

H(5A) 7225 11615 8456 45

H(3A) 3365 14363 9215 60

H(9A) 5359 7310 9761 47

H(17A) 5233 10251 7320 46

H(2A) 2010 12837 9721 55

H(10A) 8209 6060 9272 46

H(4A) 6095 13769 8599 56

_________________________________________________________

Table 6: Torsion angles [°] for **1**.

| C(11)-C(12)-C(13)-C(18)  C(7)-C(12)-C(13)-C(18)  C(11)-C(12)-C(13)-C(14)  C(7)-C(12)-C(13)-C(14)  C(7)-C(12)-C(11)-C(10)  C(13)-C(12)-C(11)-C(10)  C(7)-C(12)-C(11)-S(2)  C(13)-C(12)-C(11)-S(2)  C(14)-S(2)-C(11)-C(10)  C(14)-S(2)-C(11)-C(12)  C(11)-C(12)-C(7)-C(8)  C(13)-C(12)-C(7)-C(8)  C(11)-C(12)-C(7)-C(6)  C(13)-C(12)-C(7)-C(6)  C(8)-C(7)-C(6)-C(5)  C(12)-C(7)-C(6)-C(5)  C(8)-C(7)-C(6)-C(1)  C(12)-C(7)-C(6)-C(1)  C(14)-C(13)-C(18)-C(17)  C(12)-C(13)-C(18)-C(17)  C(16)-C(15)-C(14)-C(13)  C(16)-C(15)-C(14)-S(2)  C(18)-C(13)-C(14)-C(15)  C(12)-C(13)-C(14)-C(15)  C(18)-C(13)-C(14)-S(2)  C(12)-C(13)-C(14)-S(2)  C(11)-S(2)-C(14)-C(15)  C(6)-C(5)-C(4)-C(3) | 165.3(2)  −11.3(4)  −6.3(3)  177.1(2)  8.6(3)  −168.6(2)  −175.22(16)  7.6(2)  170.7(2)  −5.38(18)  −12.5(3)  164.0(2)  165.3(2)  −18.3(4)  165.7(2)  −12.2(4)  −6.3(3)  175.8(2)  −4.7(3)  −175.9(2)  −3.0(3)  172.96(19)  6.0(3)  178.8(2)  −170.40(17)  2.4(2)  −174.7(2)  0.5(4) | C(11)-S(2)-C(14)-C(13)  C(14)-C(15)-C(16)-C(17)  C(5)-C(6)-C(1)-C(2)  C(7)-C(6)-C(1)-C(2)  C(5)-C(6)-C(1)-S(1)  C(7)-C(6)-C(1)-S(1)  C(8)-S(1)-C(1)-C(2)  C(8)-S(1)-C(1)-C(6)  C(12)-C(7)-C(8)-C(9)  C(6)-C(7)-C(8)-C(9)  C(12)-C(7)-C(8)-S(1)  C(6)-C(7)-C(8)-S(1)  C(1)-S(1)-C(8)-C(7)  C(1)-S(1)-C(8)-C(9)  C(1)-C(6)-C(5)-C(4)  C(7)-C(6)-C(5)-C(4)  C(7)-C(8)-C(9)-C(10)  S(1)-C(8)-C(9)-C(10)  C(13)-C(18)-C(17)-C(16)  C(15)-C(16)-C(17)-C(18)  C(4)-C(3)-C(2)-C(1)  C(6)-C(1)-C(2)-C(3)  S(1)-C(1)-C(2)-C(3)  C(8)-C(9)-C(10)-C(11)  C(12)-C(11)-C(10)-C(9)  S(2)-C(11)-C(10)-C(9)  C(2)-C(3)-C(4)-C(5) | 1.61(18)  −1.4(4)  6.2(4)  179.3(2)  −170.12(18)  3.0(3)  −175.4(2)  0.77(19)  7.9(3)  −170.3(2)  −174.79(16)  7.0(3)  −4.58(19)  172.7(2)  −4.7(4)  −176.3(2)  1.6(4)  −175.43(18)  0.5(4)  2.7(4)  −1.2(4)  −3.3(4)  172.5(2)  −6.0(3)  0.8(4)  −174.94(18)  2.6(4) |
| --- | --- | --- | --- |
